# Iron overload alters the energy metabolism in patients with myelodysplastic syndromes: results from the multicenter FISM BIOFER study

**DOI:** 10.1038/s41598-020-66162-y

**Published:** 2020-06-08

**Authors:** Daniela Cilloni, Silvia Ravera, Chiara Calabrese, Valentina Gaidano, Pasquale Niscola, Enrico Balleari, Daniela Gallo, Jessica Petiti, Elisabetta Signorino, Valentina Rosso, Cristina Panuzzo, Federica Sabatini, Giacomo Andreani, Matteo Dragani, Carlo Finelli, Antonella Poloni, Monica Crugnola, Maria Teresa Voso, Susanna Fenu, Annamaria Pelizzari, Valeria Santini, Giuseppe Saglio, Marina Podestà, Francesco Frassoni

**Affiliations:** 1 Department of Clinical and Biological Sciences, University of Turin, Turin, Italy; 2 Stem Cell and Cellular Therapy Laboratory, Institute G. Gaslini, Genova, Italy; 3 Department of Experimental Medicine, University of Genova, Genova, Italy; 4 Department of Haematology, S. Eugenio Hospital, Rome, Italy; 5 Department of Haematology and Oncology, IRCCS AOU San Martino - IST, Genova, Italy; 6 Department of Haematology, S. Orsola-Malpighi Hospital, Bologna, Italy; 7 Division of Hematology, Ospedali Riuniti, Ancona, Italy; 8 Division of Hematology, Azienda Ospedaliero-Universitaria di Parma, Parma, Italy; 9 Department of Biomedicine and Prevention, Universita’ Tor Vergata, Rome, Italy; 10 Haematology Department, San Giovanni-Addolorata Hospital, Rome, Italy; 11 UO Ematologia, Ospedali Civili, Brescia, Brescia, Italy; 12 Department of Experimental and Clinical Medicine, Università degli Studi di Firenze, Florence, Italy

**Keywords:** Myelodysplastic syndrome, Cell biology, Oncology

## Abstract

Myelodysplastic syndromes (MDS) are hematological malignancies characterized by ineffective hematopoiesis and increased apoptosis in the bone marrow, which cause peripheral cytopenia. Mitochondria are key regulators of apoptosis and a site of iron accumulation that favors reactive oxygen species (ROS) production with detrimental effects on cell survival. Although the energy metabolism could represent an attractive therapeutic target, it was poorly investigated in MDS. The purpose of the study was to analyze how the presence of myelodysplastic hematopoiesis, iron overload and chelation impact on mitochondrial metabolism. We compared energy balance, OxPhos activity and efficiency, lactic dehydrogenase activity and lipid peroxidation in mononuclear cells (MNCs), isolated from 38 MDS patients and 79 healthy controls. Our data show that ATP/AMP ratio is reduced during aging and even more in MDS due to a decreased OxPhos activity associated with an increment of lipid peroxidation. Moreover, the lactate fermentation enhancement was observed in MDS and elderly subjects, probably as an attempt to restore the energy balance. The biochemical alterations of MNCs from MDS patients have been partially restored by the *in vitro* iron chelation, while only slight effects were observed in the age-matched control samples. By contrast, the addition of iron chelators on MNCs from young healthy subjects determined a decrement in the OxPhos efficiency and an increment of lactate fermentation and lipid peroxidation. In summary, MDS-MNCs display an altered energy metabolism associated with increased oxidative stress, due to iron accumulation. This condition could be partially restored by iron chelation.

## Introduction

Myelodysplastic syndromes (MDS) are a heterogeneous group of diseases, characterized by a clonal and ineffective hematopoiesis, an increased apoptosis within the bone marrow and risk of transformation into acute myeloid leukemia (AML)^1^. MDS affect especially the elderly and are characterized by one or more cytopenias, mainly anemia, often requiring red blood cell transfusions, potentially leading to iron overload. Retrospective studies suggest that iron overload, besides organ damage, decreases survival and increases the risk of evolution towards AML, although prospective data are lacking^2,3^.

Oxidative phosphorylation (OxPhos) is the metabolic pathway devoted to the ATP aerobic production^4^. Many catabolic biochemical processes, such as glycolysis, the citric acid cycle and, indirectly, beta oxidation, produce the reduced coenzymes NADH and FADH_2_, necessary to sustain the OxPhos activity^5^. In particular, the NADH and FADH_2_ electrons pass to oxygen, the final acceptor, through a series of respiratory complexes, which form the electron transport chain, located in the inner membrane of the mitochondrion. This system in coupled with formation of a proton gradient across the inner mitochondria membrane, which represents the driving force to produce ATP. When the oxygen consumption is perfectly coupled with the ATP synthesis, the system is very efficient and the risk of reactive oxygen species (ROS) production is limited^6^. By contrast, when oxygen consumption is not finalized to energy production, the ROS production increases, determining oxidative stress^7,8^. Moreover, other mitochondrial functions, such as thermoregulation, regulation of apoptosis/cell proliferation, detoxification of dangerous compounds, heme and iron sulfur cluster biosynthesis, regulation of cellular redox state and regulation of several second messengers, are involved in the production of free radicals, concurring to the increment of cellular oxidative stress. Among ROS, the superoxide radical (·O_2_^-^), which derives from the first single-electron reduction of molecular oxygen, displays low reactivity and toxicity, but may function as an important second messenger in the cell. ·O_2_^-^ is then reduced to hydrogen peroxide (H_2_O_2_), which is highly reactive and reacts with partially reduced metal ions such as Fe^3+^ and Cu^2+^, producing an extremely powerful oxidant, the hydroxyl radical (OH·), via the Fenton’s reaction^9,10^.

Increased production of ROS is the hallmark of many tumors including myelodysplastic syndromes^11^. ROS are capable of damaging proteins, lipids and DNA. Although high levels of ROS promote DNA mutations and genetic instability, over the last 20 years a more nuanced view of the role of ROS in cancer has come to light. Specifically, cancer cells generate increased ROS that are capable of increasing tumorigenesis by activating signaling pathways that regulate cellular proliferation, metabolic alterations, and angiogenesis^12,13^. However, from a metabolic point of view, cancer cells are characterized by a fermentative glycolytic metabolism, even when oxygen is available (Warburg effect^14^). This increment of glucose intake appears not only as a source of energy production^15^ but also as a basis to obtain the building blocks necessary for rapid proliferation^14^.

Since, mitochondria dysfunction, oxidative stress, and iron overload appears as emerging key players of MDS pathogenesis and progression, the purpose of this study is to analyze the MDS energetic metabolism and its link with the oxidative stress and the iron overload. In particular, we investigated aerobic ATP production, glycolysis, mitochondrial efficiency and lipid peroxidation of mononuclear cells (MNCs) isolated from MDS patients, comparing the results obtained on MNCs from young and elderly healthy subjects. Moreover, we analyzed the same parameters after treatment with two iron chelators.

## Results

### Cellular energy status decreases in MDS mononuclear cells

**Figure 1:**
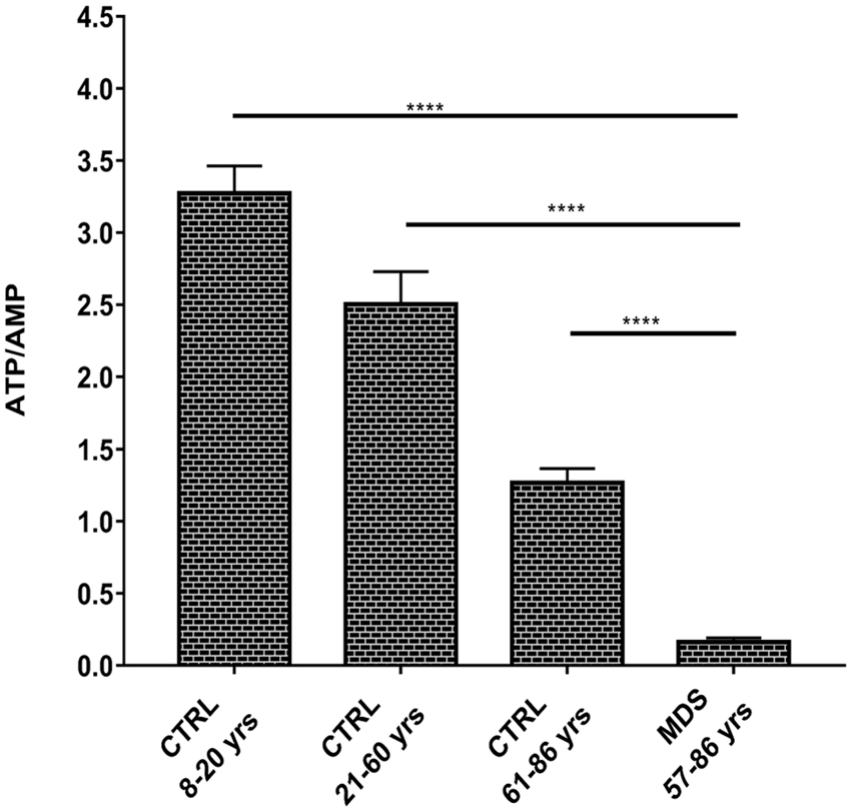
ATP/AMP ratio. ATP/AMP ratio in MNCs isolated from young healthy subjects (CTRL 8–20 yrs), adult healthy subjects (CTRL 21–60 yrs), elderly healthy subjects (CTRL 61–86 yrs) and MDS patients with iron overload. Each column represents the mean ± SEM. Data are analyzed by one-way ANOVA followed by Tukey’s multiple comparison test. **** indicates a significant difference for p < 0.0001 between MDS sample and the healthy controls.

The cellular energy status has been investigated in term of ATP/AMP ratio, which represents the balance between the available energy (ATP) and the utilized energy (AMP). The measured ATP derives both from anaerobic glycolysis and OxPhos metabolism. As reported in Fig. 1, ATP/AMP ratio progressively decreases from young controls (8–20 yrs) to adult controls (21–60 yrs) and to elderly controls (61–86 yrs). Interestingly, MDS subjects show an additional decrement of this ratio, indicating a further reduction of the energetic availability (young CTRL vs elderly CTRL p < 0.0001 and elderly CTRL vs MDS p < 0.0001). More in details, the ATP/AMP mean ratio ± SEM in young CTRL is 3.3 ± 0.2, in adult CTRL is 2.5 ± 0.2 and in elderly CTRL is 1.3 ± 0.1, while it is 0.2 ± 0.03 in MDS subjects.

### OxPhos is defective in MDS mononuclear cells

To evaluate whether the decrement of ATP/AMP ratio in elderly and MDS subjects is due to a dysfunction of mitochondrial energy metabolism, we analyzed the OxPhos activity. Oxygen consumption have been assayed after stimulation with pyruvate/malate (P/M) or succinate, to stimulate the pathways composed by complexes I, III and IV or complexes II, III and IV, respectively.

**Figure 2:**
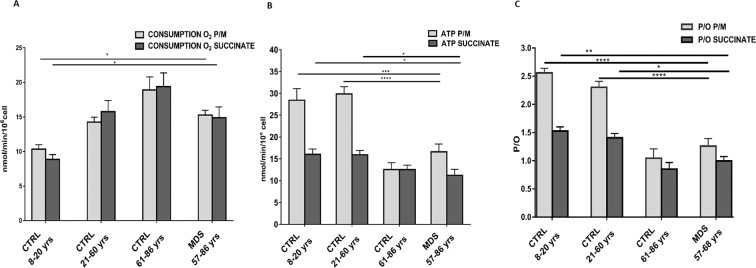
Oxygen consumption, ATP synthesis and P/O value in the presence of pyruvate/malate or succinate. Panel A: Oxygen consumption after stimulation with pyruvate/malate (P/M) (light grey column) or succinate (dark gray) in MNCs isolated from young healthy subjects (CTRL 8–20 yrs, n = 18), adult healthy subjects (CTRL 21–60 yrs; n = 18), elderly healthy subjects (CTRL 61–86 yrs, n = 25) and MDS patients with iron overload (n = 22). Panel B: aerobic ATP synthesis after stimulation with P/M (light grey column) or succinate (dark gray) in MNCs isolated from young healthy subjects (CTRL 8–20 yrs, n = 18), adult healthy subjects (CTRL 21–60 yrs; n = 18), elderly healthy subjects (CTRL 61–86 yrs, n = 25) and MDS patients with iron overload (n = 22). Panel C: P/O ratio, as marker of OxPhos efficiency, in MNCs isolated from young healthy subjects (CTRL 8–20 yrs, n = 18), adult healthy subjects (CTRL 21–60 yrs; n = 18), elderly healthy subjects (CTRL 61–86 yrs, n = 25) and MDS patients with iron overload (n = 22). For each Panel, data are expressed as mean ± SEM. Data are analyzed by one-way ANOVA followed by Tukey’s multiple comparison test. *, **, ***, **** indicate a significant difference for p < 0.05, p < 0.01, p < 0.001, p < 0.0001, respectively, between MDS sample and the healthy controls.

As shown in Fig. 2 Panel A, the oxygen consumption increases progressively with age, and the respiration of MDS-MNCs appears no-significantly different from those of adult and elderly controls. Conversely, ATP synthesis is significantly reduced in elderly control and, less marked, in MDS with respect to young and adult, especially for the system composed by complexes I, III and IV (Fig. 2, Panel B), which is the most efficient pathway, but also the main ROS producer^10^. In details, the oxygen consumption, expressed as mean ± SEM, after stimulation with pyruvate/malate is 10.3 ± 0.7 in young CTRL, 14.3 ± 0.7 in adult CTRL, 18.9 ± 1.9 in elderly CTRL, and it is 15.3 ± 0.7 in MDS subjects. The oxygen consumption after stimulation with succinate is 8.8 ± 0.7 in young CTRL, 15.8 ± 1.6 in adult CTRL, 19.4 ± 1.9 in elderly CTRL, and 15.7 ± 1.7 in MDS subjects. Regarding the ATP synthesis, after stimulation with pyruvate/malate we have observed an ATP production of about 28.5 ± 2.7 in young CTRL, 29.9 ± 2.7 in adult CTRL, 12.6 ± 1.5 in elderly CTRL, and 16.6 ± 1.8 in MDS subjects; and after stimulation with succinate 16.0 ± 1.2 in young CTRL, 15.9 ± 1 in adult CTRL, 12,5 ± 1 in elderly CTRL, and 11.2 ± 1.4 in MDS subjects.

In order to obtain a more precise picture of the mitochondrial efficiency, we calculated the P/O value, as the ratio between the produced ATP and the oxygen consumption (Fig. 2, Panel C). The P/O ratio is clearly reduced in elderly CTRL and MDS compared to young or adult CTRL. In particular, the P/O ratio in young CTRL is 2.6 ± 0.1 for P/M and 1.5 ± 0.1 for succinate (similarly to the values reported in literature^6^), while in the other groups decreases parallel with the age: 2.3 ± 0.1 for adult CTRL, 1 ± 0.2 for elderly CTRL, 1.3 ± 0.1 for MDS, after P/M induction, and 1.4 ± 0.1 for adult CTRL, 0.9 ± 0.1 for elderly CTRL, 1 ± 0.1 for MDS, after succinate stimulation. Notably, the difference is again more pronounced in the presence of P/M compared to that obtained after succinate induction.

### Lactate fermentation is enhanced in MDS mononuclear cells

**Figure 3:**
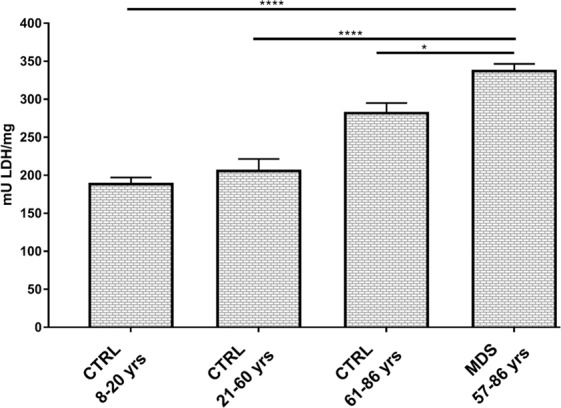
LDH activity assay. LDH activity has been evaluated as marker of anaerobic glycolysis in MNCs isolated from young healthy subjects (CTRL 8–20 yrs, n = 18), adult healthy subjects (CTRL 21–60 yrs; n = 20), elderly healthy subjects (CTRL 61–86 yrs, n = 25) and MDS patients with iron overload (n = 16). Each column represents the mean ± SEM. Data are analyzed by one-way ANOVA followed by Tukey’s multiple comparison test. * indicates a significant difference for p < 0.05 between MDS and CTRL 61–86 yrs; **** indicates a significant difference for p < 0.0001 between MDS sample and the CTRL 8–20 or the CTRL 21–60.

In order to evaluate the contribution of the anaerobic glycolysis to the cell energy status, we evaluated the lactate dehydrogenase (LDH) activity, which converts pyruvate to lactate, when oxygen is absent or in short supply. In our samples, LDH activity is higher in MDS and elderly CTRL, as compared to young and adult controls (p < 0.0001) (Fig. 3). More in details, in young CTRL the mean LDH activity ± SEM is 188.5 ± 8.7 mU/mg, in adult CTRL is 205.6 ± 15.9 mU/mg, in elderly CTRL is 281.7 ± 13.4 mU/mg and in MDS is 337 ± 9.6 mU/mg.

### Oxidative stress is increased in mononuclear cells from MDS patients

**Figure 4:**
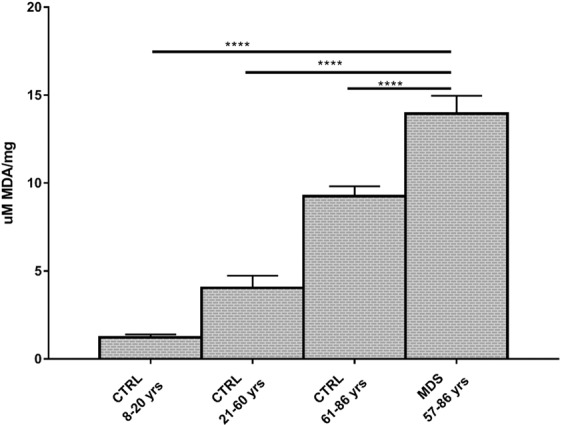
Lipid peroxidation evaluated through the malondialdehyde levels. Malondialdehyde (MDA) levels have been evaluated in MNCs isolated from young healthy subjects (CTRL 8–20 yrs, n = 18), adult healthy subjects (CTRL 21–60 yrs; n = 24), elderly healthy subjects (CTRL 61–86 yrs, n = 28) and MDS patients with iron overload (n = 30). Each column represents the mean ± SEM. Data are analyzed by one-way ANOVA followed by Tukey’s multiple comparison test. **** indicates a significant difference for p < 0.0001 between MDS sample and the heathy controls.

Since the OxPhos inefficiency is linked to an increment of oxidative stress production, we have evaluated the MDA level, as a marker of lipid peroxidation. MDA levels increase in elderly controls compared to young controls and appear even higher in MDS samples: p < 0.001 for MDS vs elderly CTRL, p < 0.0001 for elderly CTRL vs adult CTRL, p < 0.0001 for adult CTRL vs young CTRL. Going into details, the mean ± SEM MDA levels are: 1.2 ± 0.2 μM/mg in young CTRL, 4 ± 0.7 μM/mg in adult CTRL, 9.3 ± 0.6 μM/mg in elderly CTRL and 13.9 ± 1 μM/mg in MDS patients (Fig. 4).

### Iron chelation partially restores the energy balance in MDS mononuclear cells

Since MDS patients display a high iron accumulation, which can contribute to the oxidative stress production and the consequent alteration of energy metabolism, we have incubated MNCs, *in vitro* for 24 hours, with two iron chelators: deferasirox (DFX) or deferoxamine (DFO).

**Figure 5:**
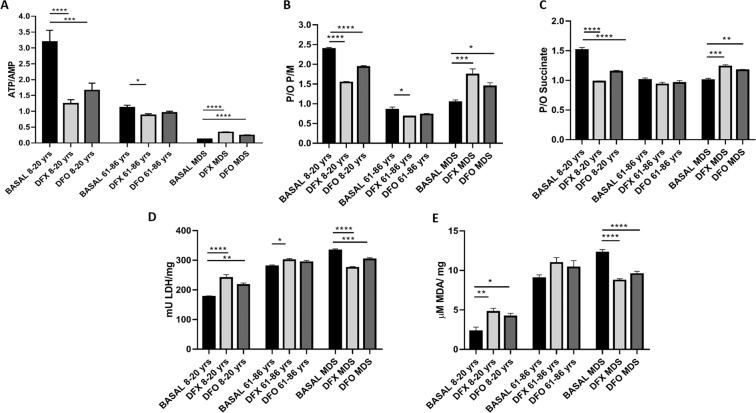
Evaluation of energy metabolism and lipid peroxidation after the treatment with iron chelators. Each Panel represents the data obtained on MNCs treated for iron chelation with deferasirox (DFX, light grey column) or deferoxamine (DFO, grey column). BASAL is for untreated samples (black column). Panel A: ATP/AMP ratio in MNCs isolated from young controls (CTRL 8–20 yrs, n = 9), elderly controls (CTRL 61–86 yrs, n = 7) and MDS patients (MDS, n = 19). Panel B: P/O ratio after stimulation with pyruvate/malate (P/M) in MNCs isolated from young controls (CTRL 8–20 yrs, n = 9), elderly controls (CTRL 61–86 yrs, n = 7) and MDS patients (MDS, n = 19). Panel C: P/O ratio after stimulation with succinate in MNCs isolated from young controls (CTRL 8–20 yrs, n = 9), elderly controls (CTRL 61–86 yrs, n = 7) and MDS patients (MDS, n = 19). Panel D: LDH activity in MNCs isolated from young controls (CTRL 8–20 yrs, n = 9), elderly controls (CTRL 61–86 yrs, n = 7) and MDS patients (MDS, n = 19). Panel E: MDA levels in MNCs isolated from young controls (CTRL 8–20 yrs, n = 9), elderly controls (CTRL 61–86 yrs, n = 7) and MDS patients (MDS, n = 19). Each column represents the mean ± SEM. Data are analyzed by one-way ANOVA followed by Tukey’s multiple comparison test. *, **, ***, **** indicate a significant difference for p < 0.05, 0.01, 0.001 or 0.0001, respectively, between the untreated sample and the MNCs treated with iron chelators.

Regarding the energy balance, DFX and DFO determined a decrease in the ATP/AMP ratio in cells from young and elderly CTRL, more evident in the age range between 8–20 years old. Conversely, the treatment induced an increment of energy availability in MNCs from MDS patients, as shown in Fig. 5 Panel A. Moreover, DFX seems to be more efficient than DFO in increasing this ratio.

More in details, the ATP/AMP ratio decreases in young CTRL from a mean value of 3.19 ± 0.37 to 1.24 ± 0.13 with DFX and 1.66 ± 0.23 with DFO (p < 0.0001 and p < 0.001, respectively). In elderly, it decreases from a mean value of 1.12 ± 0.08 to 0.87 ± 0.05 with DFX and 0.95 ± 0.06 with DFO (p < 0.05 only for DFX). By contrast, in MDS the ATP/AMP ratio increases significantly from 0.12 ± 0.01 to 0.33 ± 0.02 with DFX and 0.24 ± 0.01 with DFO (p < 0.0001 for both).

The negative effect on young healthy subjects could depend by the chelation of the iron necessary for the normal mitochondrial function. On the contrary, in MDS the iron chelation improved the mitochondrial activity highly damaged by excessive iron. This effect was extremely consistent from one sample to another (data not shown).

### OxPhos improves in MDS after iron chelation

As expected, the improvement of the ATP/AMP ratio in MDS patients after iron chelation is associated to the increased efficiency of the mitochondrial respiration. After incubation with DFX or DFO, oxygen consumption decreased in MDS patients, after stimulation either with P/M or succinate, while ATP synthesis increased (Supplementary Fig. 1), indicating a better coupling of OxPhos machinery. This hypothesis is confirmed by the improvement of the P/O ratio in MDS with both DFX and DFO after stimulation with P/M or succinate. Conversely, the P/O ratio in the presence of two chelators decreased in healthy controls, justifying the impairment of the ATP/AMP ratio reported above. Interestingly, both the negative and the positive effect of chelators treatment are more evident on the OxPhos metabolism induced by pyruvate/malate. More in details, the P/M P/O ratio decreases in young CTRL from 2.39 ± 0.04 to 1.54 ± 0.03 with DFX and 1.93 ± 0.03 with DFO (p < 0.0001 for both). In elderly healthy subjects, it decreases from a mean value of 0.85 ± 0.06 to 0.68 ± 0.02 with DFX and 0.73 ± 0.03 with DFO (p < 0.05 only for DFX). In MDS, the P/M P/O ratio increases significantly from 1.04 ± 0.06 to 1.74 ± 0.14 with DFX and 1.44 ± 0.09 with DFO (p < 0.001 and p < 0,05, respectively). Regarding the succinate P/O ratio, the value in young subjects passes from 1.51 ± 0.05 to 0.98 ± 0.01 with DFX and 1.15 ± 0.02 with DFO (p < 0.0001 for both). In the elderly subjects, it passes from 1.01 ± 0.03 to 0.93 ± 0.04 with DFX and 0.96 ± 0.04 with DFO (no significant differences). In MDS MNCs, the P/O value increases from 1.01 ± 0.03 to 1.23 ± 0.03 with DFX and 1.17 ± 0.01 with DFO (p < 0.001 and p < 0.01, respectively).

### Anaerobic glycolysis decreases after iron chelation

As shown in Fig. 5, Panel D, after incubation with iron chelators, MNCs from MDS patients show a significant decrease in LDH activity (p < 0.0001 for DFX and p < 0.001 for DFO). Interestingly the reduction of LDH activity is more pronounced with DFX than DFO (p < 0.0001).

By contrast, LDH activity is marked increase in young control (p < 0.0001 for DFX and 0.01 for DFO) and shows a slight enhancement in elderly CTRL, significant only for DFX treatment (p < 0.05).

More in details, the LDH activity decreases in young CTRL from 176.3 ± 3.7 to 240.4 ± 11.4 with DFX and 216.7 ± 6.6 with DFO. In elderly healthy subjects, the basal activity is 279.3 ± 4.7 and shifts to 300.2 ± 5.6 with DFX and 293.4 ± 6.6 with DFO. In MDS MNCs, the basal LDH activity is 332.7 ± 5.8, and passes to 274.6 ± 4 with DFX and 303 ± 5.4 with DFO.

### Lipid peroxidation decreases after iron chelation in MDS

Analyzing the lipid peroxidation in response to iron chelating treatment, we have observed a significant decrement of MDA levels (p < 0.0001 for both DFX and DFO) (Fig. 5, Panel E). By contrast, there was a trend towards increasing MDA levels in healthy controls, although statistically significant only for young subject (p < 0.001 for DFX and p < 0.05 for DFO), probably because iron chelators induced an indirect damage to the respiratory chain by removing the necessary iron.

More in details, the MDA in young CTRL is 2.3 ± 0.5, 4.76 ± 0.4 with DFX and 4.2 ± 0.4 with DFO. In elderly healthy subjects, the basal value is 9.0 ± 0.4 and passes to 10.9 ± 0.7 with DFX and 10.4 ± 0.9 with DFO. In MDS MNCs, MDA basal value is 12.3 ± 0.4, 8.7 ± 0.2 with DFX and 9.5 ± 0.3 with DFO.

### Correlation between mitochondrial dysfunction, disease characteristics and systemic iron overload

Finally, we tried to correlate our results both with the disease features and with the markers of systemic iron overload. In particular, we investigated hemoglobin (Hb) levels, bone marrow blast percentage, and R-IPSS. No significant correlation is found between our data and the parameters mentioned above for pre-treatment conditions. In particular, cellular ATP deficiency, lactate dehydrogenase activity, mitochondrial function and MDA levels are not associated with any disease feature. In addition, we have looked for links between our data and ferritin or transferrin saturation, finding a strong correlation between MDA and serum ferritin levels with a Pearson product-moment correlation coefficient (PCC) of 0.8 (data not shown).

## Discussion

Myelodysplastic syndromes are a heterogeneous group of diseases characterized by increased apoptosis in the bone marrow. Many molecular alterations have been implicated in MDS pathogenesis^16^. However, most of them resulted in increased apoptosis and ineffective erythropoiesis in the bone marrow. Mitochondria could play a crucial role, being the key regulators of apoptosis. Several lines of evidences linked mitochondrial dysfunction to dyserythropoiesis, including animal models carrying mitochondrial DNA mutations^17^. In addition, mitochondrial DNA has been found to be frequently mutated in MDS^18^ and also nuclear-encoded mitochondrial proteins have been found to be altered^19^. Mitochondria are also sites of iron accumulation, a process that frequently occurs in MDS and that favors ROS production. ROS, in turn, can have detrimental effects on cell survival: they increase lipid peroxidation and organelle damage, leading to cell death^20^, but also can damage DNA, favoring genomic instability, which may contribute to leukemic evolution. Finally, and most importantly for our study, mitochondria are considered the principal source of energy, being the site of OxPhos. Energy metabolism is known to be altered in cancer cells and it could represent an attracting therapeutic target, but little is known about energy metabolism in hematological malignancies, and especially in MDS. Schildgen *et al*.^21^ indirectly addressed this issue, demonstrating that the transcription rate of four pivotal subunits of the mitochondrial respiratory chain in myelodysplastic CD34 + cells was reduced, and that the stoichiometry of mitochondrial mRNA was altered. However, at the best of our knowledge, no study has ever directly evaluated the energy metabolism of MDS cells. Therefore, we analyzed in detail the MDS energy metabolism, trying to link this aspect to oxidative stress and iron overload. In particular, we compared energy balance, anaerobic glycolysis rate, OxPhos activity and efficiency, and lipid peroxidation in MNCs from MDS patients, comparing the results to those obtained on healthy controls. Results show that, although the ATP/AMP ratio decreased proportionally to the aging, MNCs from MDS patients displayed an energy status lower in comparison to that of age-matched healthy subjects, suggesting an alteration in the energy balance. To understand the origins of this energy shortage, we analyzed the OxPhos metabolism induced by P/M or succinate, observing an increment of oxygen consumption, but a decrement of ATP synthesis, both in elderly healthy subjects and MDS patients in comparison to the young controls. This causes the progressive impairment of the OxPhos efficiency, as indicated by the decrement of P/O value, both after P/M or succinate induction. This determines not only a reduction in energy synthesis, but also an increment in the oxidative stress, since the uncoupled respiration is associated to the reactive oxygen species (ROS) production, especially from complex I. Moreover, the impairment of aerobic energy metabolism is associated with an increment of lactate dehydrogenase (LDH) activity, both in MDS patients and in elderly subjects with respect to the younger control, suggesting an attempt to restore the energy balance.

Interestingly, despite the P/O values appear similar in MDS and elderly healthy subjects, MDS-MNCs display a very low energy status, which can be only partially justify by the OxPhos impairment. This apparent discrepancy could be explained by the high energy expenditure necessary to contrast the increment of oxidative stress production, which characterized the MDS cells^22^.

On the other hand, our data demonstrate a higher lipid peroxidation in MDS samples in comparison to the age-matched control samples, which seem associated with the iron overload since the MDA level decreases significantly after the incubation with iron chelators. However, the low ATP/AMP ratio in MDS cells could be also due to an unbalance between the enzymatic activities that convert AMP in ADP and ATP, and vice versa, such as the adenylate kinase family^23^ or by the increment of inflammatory processes associated to the iron overload. Further experiments will be necessary to investigate this topic^24^.

The involvement of iron overload in the unbalance of energy metabolism is confirmed by the positive effects on MDS-MNCs after the *in vitro* treatment with two different iron chelators, deferasirox and deferoxamine. In particular, iron chelators were able to improve the ATP/AMP ratio, to restore partially the mitochondrial function, and to reduce the MDA level. Interestingly, the effects of iron chelation on healthy controls were opposite, especially for younger subjects, provoking a reduction of mitochondrial activity and ATP/AMP ratio, probably because the chelation removed the iron necessary for the activity of respiratory complexes cytochromes. Finally, no significant correlation was found between clinical parameters and energy metabolism except one: serum ferritin levels correlated with MDA production thus confirming the detrimental role of iron overload in lipid peroxidation.

In summary, our data show that MDS cells display an altered mitochondrial metabolism associated with an uncontrolled oxidative stress production, which determines a strong energy deficiency. This seems correlated with the iron overload, as suggested by the partial restoring of the MDS-MNCs energy metabolism after iron chelation treatment.

## Material and Methods

The FISM-BIOFER 12 study has been approved by the local ethic committee San Luigi Gonzaga (number of approval: 14/2013). All methods were performed in accordance with the relevant guidelines and regulations. Ten centers participated into the study. This prospective study enrolled MDS patients with transfusion dependent iron overload. Peripheral Blood (PB) samples were collected at the time of starting iron chelation therapy (ICT). After written informed consent, PB samples from 38 MDS patients, and 79 healthy controls have been collected. Not every sample could be analyzed for all the experiments described below, mainly because of peripheral cytopenia.

Normal controls (CTRL) are divided into the following age groups: 22 young controls (mean age 12 yrs ± 2.9), 24 adult controls (mean age 38 yrs ±12.2) and 33 elderly controls (mean age 73 yrs ±8.4). MDS risk was classified according to the Revised International Prognostic Scoring System (R-IPSS)^24^. According to the WHO classification, the MDS group included: 20 MDS with single lineage dysplasia (MDS-SLD), 7 MDS with multilineage dysplasia (MDS-MLD), 7 refractory anemia with excess of blasts type I (RAEB-I), 3 RAEB II and 1 case of isolated 5q-. All MDS sample came from patients with high level of iron before being included in a protocol with iron chelation treatment. The general characteristics of these patients are summarized in Table 1. The mean age of the MDS patients was 75 ± 6.8.

**Table 1:** Clinical characteristics of the MDS patients.

UPN	Diagnosis	age	R-IPSS	Cytogenetic	serum ferritin
1	MDS-MLD	80	low	normal	2400
2	MDS-MLD	79	int	normal	1460
3	MDS-MLD	67	low	normal	280
4	MDS-MLD	78	int	trisomy 8	2800
5	MDS-MLD	80	low	normal	5220
6	MDS-MLD	79	low	normal	4870
7	MDS-MLD	69	int	del(7q)	890
8	MDS-EB-2	79	very high	del(7q)	4800
9	MDS-EB-2	84	very high	normal	NA
10	MDS-EB-1	74	high	del(20q)	1450
11	MDS-EB-1	81	int	normal	1090
12	MDS-EB-1	76	NA	NA	12800
13	MDS-EB-1	77	high	normal	2760
14	MDS-EB-1	77	int	normal	1700
15	MDS-EB-1	76	Int	normal	6670
16	MDS-SLD	80	low	normal	6520
17	MDS-SLD	85	low	normal	2400
18	MDS-SLD	74	low	normal	3380
19	MDS-SLD	61	int	del(7q)	NA
20	MDS-SLD	65	low	normal	2230
21	MDS-SLD	86	low	normal	7400
22	MDS-SLD	75	int	trisomy 8	1800
23	MDS-SLD	77	int	normal	4700
24	MDS-SLD	77	int	−7	NA
25	MDS-SLD	67	NA	NA	5240
26	MDS-SLD	75	low	normal	7400
27	MDS-SLD	73	very low	del(11q)	1700
28	MDS-SLD	80	low	normal	4000
29	MDS-SLD	83	NA	NA	6440
30	MDS-SLD	69	low	normal	2100
31	MDS-SLD	72	low	normal	1420
32	MDS-SLD	68	NA	NA	2270
33	MDS-SLD	68	verylow	normal	1380
34	del(5q)	57	very low	del(5q)	3670
35	MDS-SLD	73	low	normal	1820
36	MDS-SLD	84	NA	NA	2770
37	MDS-EB-1	82	high	normal	1889
38	MDS-EB-2	65	Very high	complex	3880

### Mononuclear cells isolation

Mononuclear cells (MNCs) were isolated from 10 ml of PB on a Ficoll gradient. All samples were sent to the laboratory and analysed within 24 hours from collection.

### Iron chelation

16 PB from healthy controls (7 young subjects with a mean age of 17 ± 3.6, 9 elderly subjects with a mean age 67 ± 5.5) and 19 myelodysplastic patients with a mean age 72 ± 8.4) were incubated with two iron chelators: Deferasirox (DFX) and Deferoxamine (DFO), for 24 hours, both at 50 uM concentration^25^.

All myelodysplastic patients have an iron overload due to blood transfusions with a serum ferritin level higher than 1000 ng/mL and a transferrin saturation higher than 70% and all of them have received at least 20 U of red blood cells. All samples were taken before starting iron chelation therapy.

### Evaluation of ATP/AMP intracellular level, lipid peroxidation and Lactate dehydrogenase (LDH) assays

ATP and AMP were measured according to the enzyme coupling method, following the NADP reduction or NADH oxidation, at 340 nm, respectively26. To assess lipid peroxidation, the malondialdehyde (MDA) level was evaluated by the thiobarbituric acid reactive substances (TBARS) test, at 532 nm^26^. LDH activity was measured as marker of anaerobic glycolysis following the NADH oxidation, at 340 nm^26^.

### Oxygen consumption measurements

Oxygen consumption was measured in a closed chamber, using an amperometric electrode (Unisense-Microrespiration, Unisense A/S, Denmark)^27^.100,000 cells, permeabilized with 0.03% digitonin for 1 min were used for each experiment. Pyruvate and malate were used to stimulate the pathway composed by complexes I, III and IV, while succinate induced the pathway formed by complexes II, III and IV. To observe the ADP-stimulated respiration rates, 0.08 mM ADP was added after pyruvate and malate or succinate addition.

### Fo F1 ATP synthase activity

ATP synthesis, trough Fo-F1 ATP synthase, was performed according to published method^28^.The reaction was monitored in a luminometer (GloMax® 20/20n Luminometer, Promega Italia, Milano, Italy), by the luciferin/luciferase chemiluminescent method, with ATP standard solutions between 10^–8^ and 10^–5^ M (luciferin/luciferase ATP bioluminescence assay kit CLSII, Roche, Basel, Switzerland).

### Evaluation of the efficiency of mitochondrial energy metabolism

The OxPhos efficiency (P/O ratio) was calculated as the ratio between the concentration of the produced ATP and the amount of consumed oxygen in the presence of respiratory substrate and ADP. When the oxygen consumption is completely devoted to the energy production, the P/O ratio should be around 2.5 and 1.5 after pyruvate + malate or succinate addition, respectively^6,28^.

### Statistical analysis

Data were analyzed by one-way ANOVA followed by Tukey’s multiple comparison test, using GraphPad Prism version 7.00. P value <0.05 was considered significant.

## Supplementary information

Supplementary 1.
