# Supplementary material for: Iron overload alters the energy metabolism in patients with myelodysplastic syndromes: results from the multicenter FISM BIOFER study
Source: Sci Rep. 2020 Jun 8;10:9156. doi: 10.1038/s41598-020-66162-y (PMC7280296; doi:10.1038/s41598-020-66162-y)
Supplement: Supplementary file 1 — Supplementary 1. [file 41598_2020_66162_MOESM1_ESM.docx]

**Iron overload alters the energy metabolism in patients with myelodysplastic syndromes: results from the multicenter FISM BIOFER study**

Daniela Cilloni,^1^* Silvia Ravera,^2,3^* Chiara Calabrese,^1^* Valentina Gaidano,^1^ Pasquale Niscola,^4^ Enrico Balleari,^5^  Daniela Gallo,^1^ Jessica Petiti, ^1^ Elisabetta Signorino,^1^ Valentina Rosso,^1^ Cristina Panuzzo, ^1^ Federica Sabatini,^2^ Giacomo Andreani, ^1^ Matteo Dragani,^1^ Carlo Finelli,^6^ Antonella Poloni,^7^ Monica Crugnola,^8^ Maria Teresa Voso,^9^ Susanna Fenu,^10^ Annamaria Pelizzari,^11^ Valeria Santini,^12^ Giuseppe Saglio, ^1^ Marina Podestà, ^2^ Francesco Frassoni.^1^


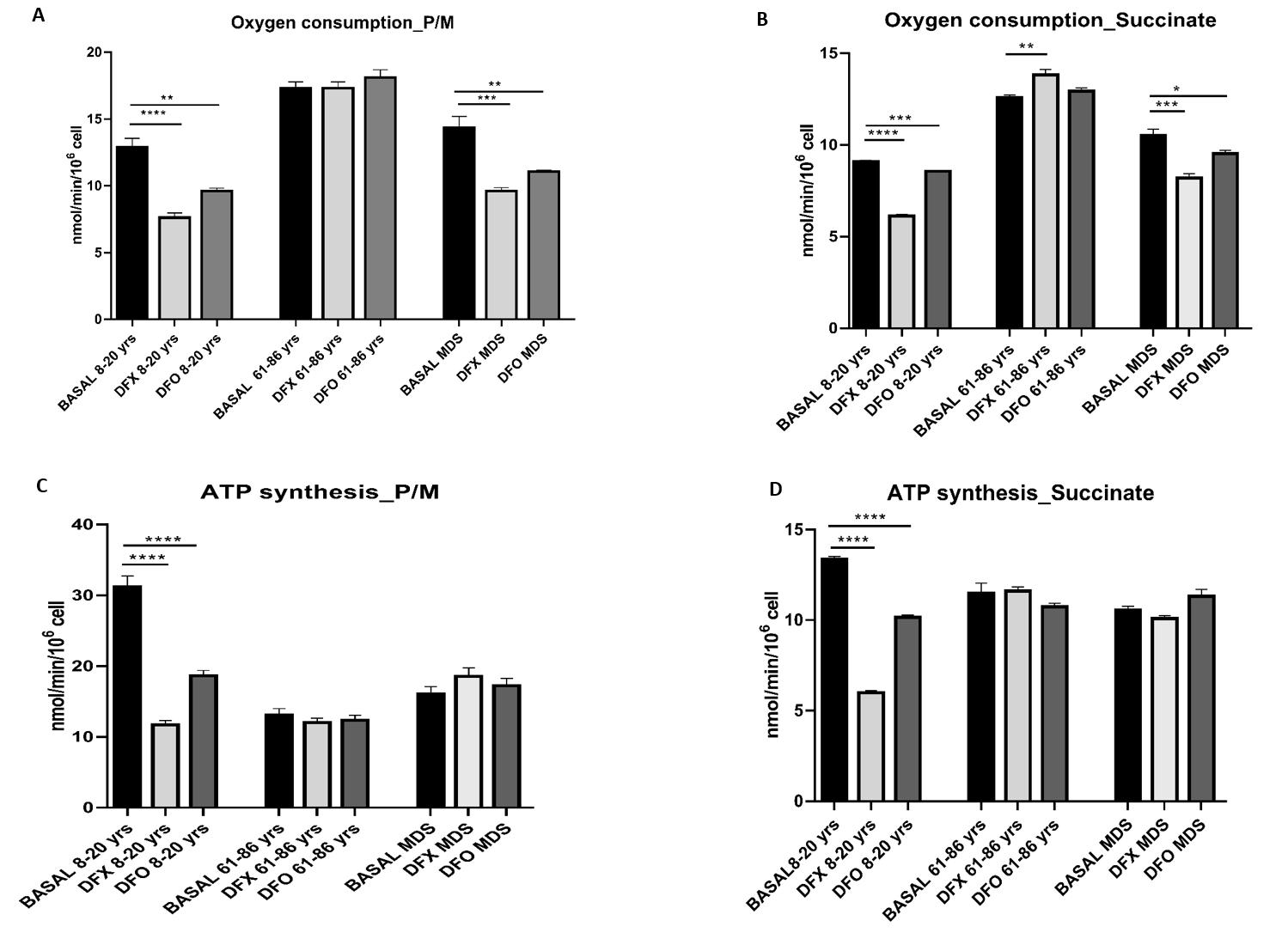


**Supplementary 1:** **Oxygen consumption and ATP synthesis after iron chelation**

Each Panel represents the data obtained on MNCs treated with deferasirox (DFX, light grey column) or deferoxamine (DFO, grey column). BASAL is for untreated samples (black column).

**Panels A and B**: Oxygen consumption after stimulation with pyruvate/malate (P/M)  or succinate, respectively, in MNCs isolated from young healthy subjects (CTRL 8-20 yrs, n=9), elderly healthy subjects (CTRL 61-86 yrs, n=7) and MDS patients with iron overload (n=19).

**Panels C and D**: aerobic ATP synthesis after stimulation with P/M or succinate, respectively, in MNCs isolated from young healthy subjects (CTRL 8-20 yrs, n=9), elderly healthy subjects (CTRL 61-86 yrs, n=7) and MDS patients with iron overload (n=19).

For each Panel, data are expressed as mean ± SEM and are analyzed by one-way ANOVA followed by Tukey's multiple comparison test. *, **, ***, **** indicate a significant difference for p<0.05, p<0.01, p<0.001, p<0.0001, respectively, between basal and treated samples.
